# Hyperspectral imaging detects perfusion and oxygenation differences between stapled and hand-sewn intestinal anastomoses

**DOI:** 10.1515/iss-2022-0007

**Published:** 2022-06-30

**Authors:** Tristan Wagner, Sonia Radunz, Felix Becker, Claire Chalopin, Hannes Kohler, Ines Gockel, Boris Jansen-Winkeln

**Affiliations:** Department of Visceral, Transplant, Thoracic and Vascular Surgery, University Hospital of Leipzig, Leipzig, Germany; Department of General, Visceral and Transplant Surgery, University Hospital of Münster, Münster, Germany; Innovation Center Computer Assisted Surgery, University of Leipzig, Leipzig, Germany; Department of General, Visceral and Vascular-Surgery, St. George’s Hospital of Leipzig, Leipzig, Germany

**Keywords:** anastomotic leakage, hand-sewn vs. stapled gastrointestinal anastomoses, hyperspectral imaging, tissue oxygenation and perfusion

## Abstract

**Objectives:**

Hand-sewn and stapled intestinal anastomoses are both daily performed routine procedures by surgeons. Yet, differences in micro perfusion of these two surgical techniques and their impact on surgical outcomes are still insufficiently understood. Only recently, hyperspectral imaging (HSI) has been established as a non-invasive, contact-free, real-time assessment tool for tissue oxygenation and micro-perfusion. Hence, objective of this study was HSI assessment of different intestinal anastomotic techniques and analysis of patients’ clinical outcome.

**Methods:**

Forty-six consecutive patients with an ileal–ileal anastomoses were included in our study; 21 side-to-side stapled and 25 end-to-end hand-sewn. Based on adsorption and reflectance of the analyzed tissue, chemical color imaging indicates oxygen saturation (StO_2_), tissue perfusion (near-infrared perfusion index [NIR]), organ hemoglobin index (OHI), and tissue water index (TWI).

**Results:**

StO_2_ as well as NIR of the region of interest (ROI) was significantly higher in stapled anastomoses as compared to hand-sewn ileal–ileal anastomoses (StO_2_ 0.79 (0.74–0.81) vs. 0.66 (0.62–0.70); p<0.001 NIR 0.83 (0.70–0.86) vs. 0.70 (0.63–0.76); p=0.01). In both groups, neither anastomotic leakage nor abdominal septic complications nor patient death did occur.

**Conclusions:**

Intraoperative HSI assessment is able to detect significant differences in tissue oxygenation and NIR of hand-sewn and stapled intestinal anastomoses. Long-term clinical consequences resulting from the reduced tissue oxygenation and tissue perfusion in hand-sewn anastomoses need to be evaluated in larger clinical trials, as patients may benefit from further refined surgical techniques.

## Introduction

The quality of intestinal anastomoses is widely considered as a key factor for short- and long-term success in abdominal surgery. Impaired wound healing, especially anastomotic leakage (AL) with consecutive severe postoperative abdominal complications, remains one of the most critical factors during surgery. AL may result in prolonged hospital stay, delayed chemotherapy and increased overall mortality, all in addition to dramatic reduced quality of life because of reoperation and necessity of stoma application. In particular, postoperative AL is associated with impaired oncological outcome [1].

The surgical anastomotic techniques have continuously been refined to attain lower complication rates and improved clinical outcomes. Nowadays, stapled anastomoses are the gold standard in anatomically difficult accessible regions and display several advantages as compared to hand-sewn approaches. Stapled anastomoses result in shortened operating time, but constitute additional costs [2], [3], [4], [5]. In easier accessible areas, e.g., stomach, small intestine, right-sided colon, and in conventional open surgery, there is no general agreement on which technique should be preferred. With regard to the occurrence of AL, hand-sewn and stapled anastomoses are equivalent in easily accessible regions [6].

Nevertheless, the impact of intraoperative intestinal micro perfusion on short- and long-term surgical outcomes in different surgical anastomotic techniques is still insufficiently understood. In image-guided and precision surgery, the relatively new method of hyperspectral imaging has demonstrated promising results for characterization of different tissues and assessment of physiologic tissue parameters [7], [8], [9], [10], [11]. HSI combines the principle of spectroscopy with digital image processing. The examined tissue is illuminated in the visual and near-infrared spectrum and the remitted spectral light waves are measured [4]. In upper gastrointestinal surgery, HSI has been turned out to be a valuable method for evaluating ischemic conditioning effects with regard to the gastric tube formation in oncologic esophagectomy [12]. In colorectal surgery, HSI has visualized the margin of colonic transection precisely [7, 8].

Hence, the objective of this study was to assess intraoperative tissue micro-perfusion and oxygenation in hand-sewn vs. stapled intestinal anastomoses using HSI.

## Material and methods

Forty-six consecutive adult patients undergoing elective surgery including ileal anastomosis after ileostomy or ileo–anal J-pouch formation following laparoscopic restorative proctocolectomy due to either colorectal cancer or ulcerative colitis were included in this prospective clinical trial. The study was approved by the local ethics committee of the medical faculty of the University of Leipzig (file number 026/18-ek) and conducted in accordance with the Helsinki Declaration of 1975, as revised in 2008.

All patients gave written informed consent to participate in the study.

The hand-sewn end-to-end anastomoses for ileostomy reversal were performed using single-layer running sutures with resorbable polydioxanone sutures in size 4/0 (Johnson & Johnson Medical GmbH, Ethicon Germany, Norderstedt) as the standard at our clinic. The side-to-side stapled anastomoses for ileo-anal J-pouch formation was performed with the 75 mm linear cutter and a blue cartridge with a 1.5 mm closed staple height (Johnson & Johnson Medical GmbH, Ethicon Germany, Norderstedt, Germany).

**Figure 1:**
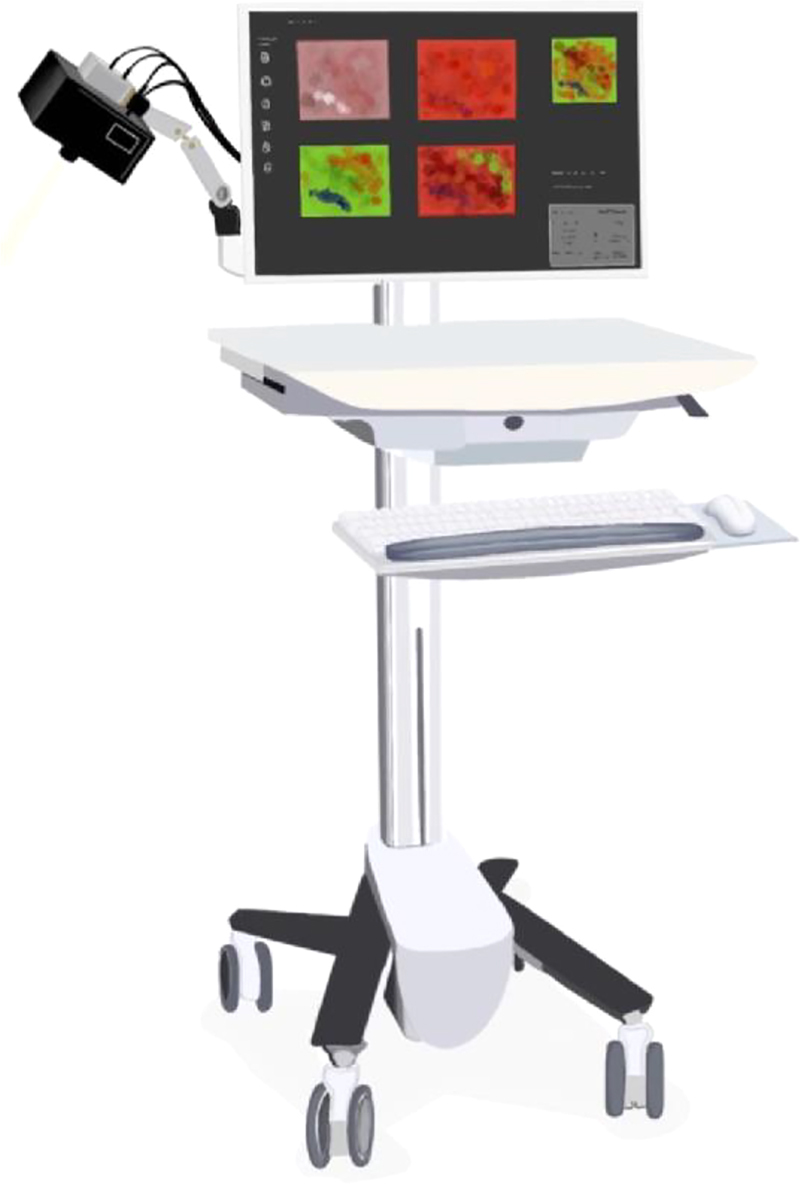
Systematical drawing of the hyperspectral camera system (HSI), TITIVA system (diaspective vision).

HSI assessment was performed after completion of the intestinal anastomoses. Images were acquired with the *TIVITA*^*®*^ *Tissue* system (Diaspective Vision GmbH, Am Salzhaff, Germany). The analysis software provides a red–green–blue image (RGB image) and four false-color images with an effective number of 640 × 480 pixels representing perfusion parameters of the recorded tissue area [4]. These parameters are tissue oxygenation (StO_2_), near-infrared (NIR) perfusion index, tissue water index (TWI), and organ hemoglobin index (OHI) [13] (Figure 1).

**Figure 2:**
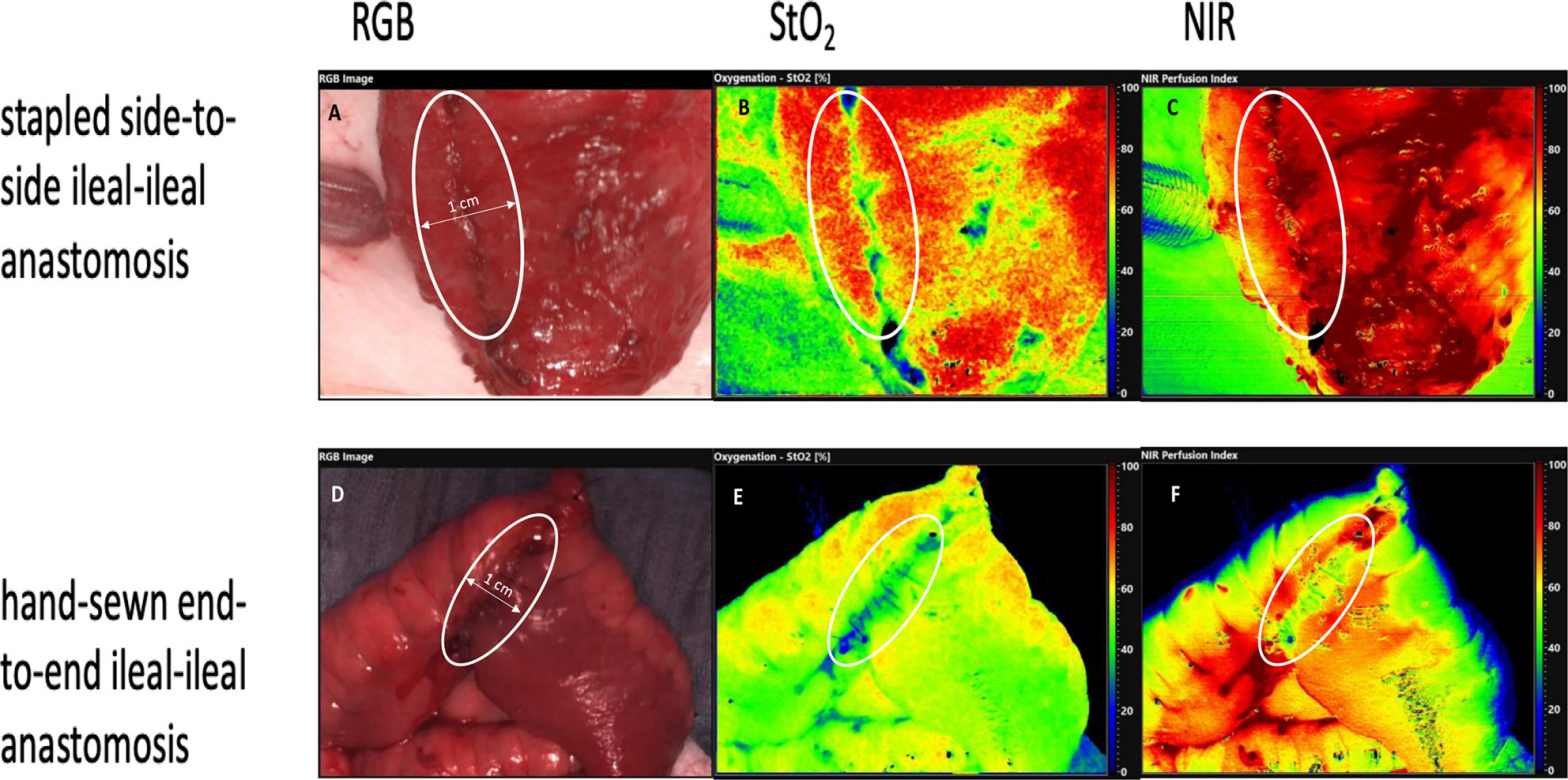
Hyperspectral imaging (HSI) of the different types of anastomoses (A/B/C stapled side-to-side ileal–ileal anastomosis, D/E/F – hand-sewn end-to-end ileal–ileal anastomosis): The analyzed ROIs are marked. RGB images are displayed on the left side (A + D) and StO_2_ (B/E) and NIR (C/F) false-color images on the right side.

A distance of exactly 50 cm between the bowel and the HSI camera was utilized for assessment of the intestinal anastomosis, and a ROI with a 0.5 cm width on both sides of the suture or staple line center was selected (Figure 2). Ambient light was turned off to ensure undisturbed image acquisition [4]. For assessment of anastomotic micro-perfusion, StO_2_ representing the relative blood oxygenation in the microcirculation of superficial tissue layers (approximately 1 mm) and NIR representing the relative oxygenation of structures in up to 4–6 mm depth were analyzed.

Data collection and statistical analyses were performed using Microsoft Excel 2013 (Microsoft Corporation, Redmond, WA, USA) and IBM SPSS Statistics Standard v24 (IBM Corporation, Chicago, USA). Data are presented as mean, median, range, and quartiles, unless stated otherwise. The t-test was performed to check for equal variances and the unpaired two-tailed Student’s t-test was used to determine statistical significance. A “p-value”≤0.05 (two-tailed) was considered statistically significant.

## Results

Median age of patients measured intraoperatively was 49 [range 18–77] years. 22 patients were females and 24 patients were males. 21 patients underwent side-to-side stapled ileal–ileal anastomosis for ileal J-pouch creation and 25 patients underwent end-to-end hand-sewn ileal–ileal anastomoses for ileostomy reversal (after short ileal segmental resection).

Underlying diseases were colorectal cancer (n=19) and ulcerative colitis (n=27). Loop ileostomies had been placed protectively in the setting of low anterior rectal resections with TME (total mesorectal excision) due to rectal cancer or laparoscopic restorative proctocolectomy for ulcerative colitis (two- or three-stage approach). Median length of hospital (LOS) stay was 9 (range 5–18) days. None of the patients in both groups developed AL or abdominal septic complications. Mortality was 0%.

StO_2_ of ROI was significantly higher in stapled anastomoses compared to hand-sewn ileal-ileal anastomoses (median: 0.79 [0.74–0.81] vs. 0.66 [0.62–0.70], p<0.001). NIR of ROI was significantly higher in stapled anastomoses compared to hand-sewn ileal–ileal anastomoses, as well (median: 0.83 [0.70–0.86] vs. 0.70 [0.63–0.76], p=0.01). OHI and TWI of ROI showed no significant differences; therefore no further investigations with those values were performed.

## Discussion

HSI has initially been applied in various clinical research areas of wound management.

Special aspects comprise monitoring of skin ulcer healing and plastic flap assessment [14], [15], [16], [17]. In oncologic esophagectomy, significant differences in StO_2_ of the gastric conduit were detected in our hands with regard to ischemic conditioning of the stomach in the context of two-step procedure with risk reduction [9]. In colorectal surgery, HSI allowed visualization of blood perfusion at resection margins to create well-perfused anastomoses [7, 8].

Our current data clearly demonstrate that the micro perfusion in the area of the bowel tissue of hand-sewn intestinal anastomoses is significantly reduced compared to the tissue in stapled anastomoses. We did not detect any postoperative complications (anastomotic leakage, abdominal septic complications, wound healing disorders, and postoperative temporary bowl obstructions) in our study population. Since the anastomoses in both groups showed no evidence of postoperative disorders, e.g., AL, abdominal sepsis/abscess, or other anastomosis related complications, we rated the perfusion/oxygenation in all anastomoses as sufficient, while tension-free construction and perfect surgical technique were preconditions in all patients, according to our internal standards. However, there are yet no established thresholds of adequate or borderline HSI-parameters in intestinal anastomoses with regard to prediction of undisturbed healing vs. AL development.

The performed technique of bowel anastomoses is influenced by the diameter of the bowel, edema, accessibility and site of anastomosis, contamination, available time and equipment, and underlying pathology [18]. In loop ileostomy reversal, the prospective randomized HASTA trial suggested that there is no significant difference between hand-sewn and stapled anastomoses with respect to AL [6]. The Cochrane Review has underlined those findings over the last decades [19]. In previous studies, the reported differences demonstrated higher costs, but a significant reduction in time of surgery when performing stapled anastomoses [2, 20, 21].

With poor overall evidence of anastomotic techniques, running sutures appear to have less perfusion than interrupted sutures [22]. Still, running sutures seem to be more common and in some studies clinically superior to single button sutures [5, 23]. However, none of these studies offer profound insights in potential differences between the two approaches with regard to physiologic parameters relevant for healing, e.g., perfusion or oxygenation. Hence, our results, for the first time in the literature according to our knowledge, hint to advantages in these specific prerequisites for regular healing in favor of stapled anastomoses vs. hand-sewn technique. Both of these most important tissue characteristics cannot be evaluated by surgeon’s visualization with his own eye intraoperatively, promoting HSI to a very promising new tool to enhance patient safety routinely in the near future.

Intestine’s perfusion can also be visualized using near-infrared fluorescence angiography with intravenously applied indocyanine green (ICG) as fluorescent dye [24, 25].

ICG has proved very valuable in strategy change and decision-making processes during surgery. The PILLAR II multicenter study showed no anastomotic leakages in patients with intraoperative ICG fluorescence angiography of blood perfusion in the anastomotic area [26]. Yet, the local resolution of this method allows blood perfusion assessment of the anastomosis only. Individual threads or even the space between the seams cannot be displayed. In contrast to HSI as presented by our data, ICG fluorescence angiography is not able to examine the fine differences between handstitched and stapled anastomoses. Furthermore, ICG bears the risk of anaphylactic reactions or other dangerous side effects due to its invasiveness. Another important aspect is that fluorescence angiography with ICG results in semiquantitative assessment and is more subject to surgeons’ individual evaluation, as contrasted to clear quantitative and objective parameters as presented by HSI.

Despite the significant findings, there are some limitations to our study. This is a single center study carrying the risk of bias for treatment. Even if the compared anastomotic techniques both require sutures of the ileum wall, these anastomoses are different in dissection of the mesenterium. Being aware that the group with a J-pouch and stapling technique and the group with ileostomy retraction and end-to-end hand sutures do not allow a direct comparison since underlying surgical expertise in those procedures, mesenterial injuries, and neoadjuvant therapies are different.

We chose side-to-side anastomoses for ileal J-pouch formation as comparison to hand-sewn end-to-end ileal–ileal anastomoses, as we do not perform other stapled anastomoses of the small intestine in elective open surgery. In our clinic, end-to-end staple-suture anastomoses are performed in left colon and rectum resections, only. Both technical groups did not develop any kind of anastomotic complication, making the interpretation of clinical outcomes (in spite of significant differences in relevant HSI-parameters) difficult. Thus, all conclusions may keep speculative, supported by the fact, that no HSI-thresholds at this time have been established for undisturbed vs. borderline vs. restricted anastomotic healing due to compromised oxygenation and perfusion of the respective segment of the small intestine.

Furthermore, we could not examine the vast majority of laparoscopic operations, as the HSI camera at the time of measurements and data collection was applicable in open procedures at the time of our clinical trial, only. This aspect has been overcome by the current availability of the new *TIVITA*^*®*^ *MINI* (for laparoscopic assessment), which has been invented in our clinic only recently.

Many confounders may occur in the analysis of perioperative outcome of intestinal anastomoses. In our small study population, the overall frequency of complications was zero; therefore, the ability to detect differences in outcome between groups and to adjust for any potential confounders is not given. Normal values and thresholds of potential anastomotic leakage with regard to the investigated perfusion parameters cannot yet be determined.

Nevertheless, this is the first study evaluating HSI assessment of intestinal anastomoses and may serve as a background for further trials in this field. Especially in risk situations, e.g., emergency surgery, mesenterial ischemia, intestinal trauma with “damage control surgery”, inflammatory bowel disease, and clinical trials are warranted.

## Conclusions

Intraoperative HSI demonstrated significant differences in tissue oxygenation and micro perfusion in hand-sewn vs. stapled intestinal anastomoses. However, due to the multifactorial background of anastomotic pathophysiology and healing, limited blood flow does not seem to be the only risk factor for anastomotic leakage. There are no “cut-off” values so far to evaluate the bowel perfusion by HSI and to predict potential anastomotic leakage. This has to be investigated in future studies.

## Supplementary Material

Supplementary Material
