# Supplementary material for: Hyperspectral imaging detects perfusion and oxygenation differences between stapled and hand-sewn intestinal anastomoses
Source: Innov Surg Sci. 2022 Jun 30;7(2):59–63. doi: 10.1515/iss-2022-0007 (PMC9574651; doi:10.1515/iss-2022-0007)
Supplement: Supplementary file 1 — Supplementary Material [file j_iss-2022-0007_suppl.pdf]

Reviewer Assessment

T. Wagner et al.: Hyperspectral imaging detects perfusion and oxygenation differences between stapled and hand-sewn intestinal anastomoses

Reviewers' Comments to Original Submission

Reviewer 1: Schiedeck, Thomas

Date received: 23-Mar-2022
Reviewer recommendation: **Accept in present form**
Reviewer overall scoring: **High**

Assessment Form scores: 5 = High/Yes; 3 = Medium/Adequate; 1 = Low

Is the subject area appropriate for the journal	5				
Does the title clearly reflect the paper's content?		4			
Does the abstract clearly reflect the paper's content		4			
Do the keywords clearly reflect the paper's content?	5				
Does the introduction present the problem clearly?		4			
Are the results/ conclusions justified?		4			
How comprehensive and up-to-date is the subject matter presented?		4			
How adequate is the data presentation?		4			
Are units and terminology used correctly?	5				
Is the number of cases adequate?			3		
Are the experimental methods/ clinical studies adequate?		4			
Is the length appropriate in relation to the content?		4			
Does the reader get new insights from the article?		4			
Please rate the practical significance.	5				
Please rate the accuracy of methods.		4			
Please rate the statistical evaluation and quality control.			3		
Please rate the appropriateness of the figures and tables.		4			
Please rate the appropriateness of the references.		4			
Please evaluate the writing style and use of language.		4			
Please judge the overall scientific quality of the manuscript.		4			
Are the methods used worthy of reproduction in greater deal?	Yes				
Would you be willing to review a revision of this manuscript?	No				

Comments to author: A very interesting method to deal with a long standing problem in visceral surgery. The authors are able to demonstrate the opportunities given by hyperspectral imaging in evaluating gastrointestinal anastomoses. I would be

interesting, to get an idea how the results would have been using Laser-immuno Fluorescense f.ex. As correctly stated by the authors anastomotical healing is an multifactorial phenomenon and regarding their results it might be the next question raised comparing running with interrupted handsewn anastomosis. All in all the method is absolutely promising. Should be accepted for publication.

Reviewer 2: anonymous

Date received: 05-Apr-2022
Reviewer recommendation: **Accept in present form**
Reviewer overall scoring: **High**

Assessment Form scores: 5 = High/Yes; 3 = Medium/Adequate; 1 = Low

Is the subject area appropriate for the journal	5				
Does the title clearly reflect the paper's content?	5				
Does the abstract clearly reflect the paper's content	5				
Do the keywords clearly reflect the paper's content?	5				
Does the introduction present the problem clearly?	5				
Are the results/ conclusions justified?			3		
How comprehensive and up-to-date is the subject matter presented?	5				
How adequate is the data presentation?			3		
Are units and terminology used correctly?			3		
Is the number of cases adequate?			3		
Are the experimental methods/ clinical studies adequate?			3		
Is the length appropriate in relation to the content?		4			
Does the reader get new insights from the article?		4			
Please rate the practical significance.		4			
Please rate the accuracy of methods.			3		
Please rate the statistical evaluation and quality control.			3		
Please rate the appropriateness of the figures and tables.		4			
Please rate the appropriateness of the references.		4			
Please evaluate the writing style and use of language.		4			
Please judge the overall scientific quality of the manuscript.			3		
Are the methods used worthy of reproduction in greater deal?	Yes				
Would you be willing to review a revision of this manuscript?	Yes				

Comments to author: The authors present a work in which they use a hyperspectral camera to measure tissue perfusion and oxygenation in small bowel anastomoses. Two groups were studied. 21 patients received an ileoanal J-pouch by stapling technique in the context of a proctocolectomy. 25 patients underwent repositioning of a double-barreled protective ileostomy with hand-sewn end-to-end anastomosis. There were no complications and no anastomotic leaks in either group. Oxygen saturation and near infrared perfusion index were significantly higher in the stapled J-pouch anastomosis group.

The work is well written and understandable. The topic is exciting and relevant to current surgical practice.
With regard to the study design, it should be noted, that the selection of a group with a J-pouch and stapling technique and a group with ileostomy retraction and end-to-end hand sutures does not allow a direct comparison. The authors are aware of this, but in my opinion this point is not enough discussed. The two groups differ more than just end-to-end vs. side-to-side

anastomosis. In the case of ileostomy, the intestine must be detached from the abdominal wall. Adhesions can lead to injuries of the meso. The intestine is dislocated in front of the abdominal wall and there is tension on the meso, so that the perfusion may not be optimal. The procedure is often performed by trainee surgeons under the supervision of a specialist. The patient's underlying disease includes all colorectal disease entities. It is often cancer patients who have received chemotherapy and radiation.
A J-pouch is usually placed during a proctocolectomy due to ulcerative colitis or FAP. The operation is actually only performed by experienced surgeons. Depending on the surgical procedure, the pouch is sometimes placed in front of the abdominal wall. However, the meso is usually not severed.
The results are presented only briefly. "Organ hemoglobin index" and "Tissue water index" are mentioned in the abstract but I can't find any corresponding results in the manuscript. How are the results presented? Are these mean values or median? When do the authors speak of range and when quartile? A table with the results might be helpful.
The fact that zero complications occurred in a total of 46 treatments is remarkable. Do the authors mean by that, that also noc wound infection or outher surgical site infection occurred? No postoperative disturbances like temporary bowl obstruction?
The pictures are good.

- Page 2, line 35: If it's a range, I would present the values like this (0.74 – 0.81); P = 0.0003 is actually p < 0.001.
- Page 7, line 47: it lacks a dot after "Salzhaff, Germany)"
- Page 8, line 22: Data presentation. What is true? Why no standard deviation or standard error when showing mean? Line 24: Is "f-test" correct or should it be "t-test". Line 29: I would write the p in "p-value" in lowercase.
- Page 11, line 47: "in contrast to...." The sentence is incomprehensible.
- Page 12, line 26: typo in "that" and line 25 in "operations"

Reviewers' Comments to the Revised Manuscript

Reviewer 2: anonymous

Date received: 03-May-2022
Reviewer recommendation: **Accept in present form**
Reviewer overall scoring: **Medium**

Assessment Form scores: 5 = High/Yes; 3 = Medium/Adequate; 1 = Low

Is the subject area appropriate for the journal	5				
Does the title clearly reflect the paper's content?	5				
Does the abstract clearly reflect the paper's content	5				
Do the keywords clearly reflect the paper's content?	5				
Does the introduction present the problem clearly?	5				
Are the results/ conclusions justified?		4			
How comprehensive and up-to-date is the subject matter presented?	5				
How adequate is the data presentation?		4			
Are units and terminology used correctly?	5				
Is the number of cases adequate?			3		
Are the experimental methods/ clinical studies adequate?			3		
Is the length appropriate in relation to the content?		4			
Does the reader get new insights from the article?	5				

Please rate the practical significance.	5				
Please rate the accuracy of methods.	5				
Please rate the statistical evaluation and quality control.	5				
Please rate the appropriateness of the figures and tables.		4			
Please rate the appropriateness of the references.		4			
Please evaluate the writing style and use of language.		4			
Please judge the overall scientific quality of the manuscript.			3		
Are the methods used worthy of reproduction in greater deal?	No				
Would you be willing to review a revision of this manuscript?	Yes				

Comments to author: All queries were resolved.

Comments by the Editor-in-Chief to Revised Submission

All reviewer comments were addressed adequately and the manuscript should be published in its present stage.
